# Gas Embolism Complicating Endoscopic Retrograde Cholangiopancreaticography: Case Report of a Complex Condition

**DOI:** 10.7759/cureus.71603

**Published:** 2024-10-16

**Authors:** Aditya K Jain, Nikita Goel, Kushagra Mathur

**Affiliations:** 1 General Medicine, Hillingdon Hospital NHS Foundation Trust, London, GBR; 2 Rheumatology, Northwick Park Hospital, Harrow, GBR; 3 General Medicine, Dartford and Gravesham NHS Trust, Dartford, GBR

**Keywords:** 2d echo, common bile duct stone, endoscopic retrograde cholangiopancreaticography, gas embolism, trans-esophageal echocardiogram

## Abstract

This case report explores the complex clinical trajectory of a 72-year-old female with a history of hypertension, iron-deficiency anaemia, and vertigo, who underwent an endoscopic retrograde cholangiopancreaticography (ERCP) procedure for common bile duct (CBD) stone removal. After an uneventful laparoscopic cholecystectomy, she continued to experience abdominal pain and icterus. Investigations including magnetic resonance cholangiopancreatography (MRCP), revealed a dilated CBD with multiple stones, prompting ERCP. During the procedure, a fall in saturation and arrhythmia were noted, leading to the diagnosis of gas embolism. Trans-esophageal echocardiography (TEE) confirmed air bubbles in cardiac chambers and a patent foramen ovale (PFO). Despite interventions, including intubation, noradrenaline infusion, and a temporary pacemaker, the patient's cardiovascular status deteriorated. Due to financial constraints, she was discharged against medical advice (DAMA) with a high-risk profile. This case highlights the rarity and iatrogenic nature of ERCP-related air embolism, emphasising the challenges in its management and underscoring the need for awareness and timely intervention. The discussion delves into the broader context of air embolism pathogenesis, referencing relevant literature and highlighting the need for continued research in managing such rare complications associated with ERCP.

## Introduction

Acute abdomen is a common presentation in the emergency department and can arise due to diverse causes, which require varied approaches to diagnosis and management. Gallbladder stones are often a contributing factor, occasionally leading to choledocholithiasis when they migrate into the common bile duct. Surgical intervention, whether open or laparoscopic, is the preferred approach for managing stones in the gall bladder depending upon the severity of symptoms. Conversely, common bile duct (CBD) stones are commonly addressed using endoscopic retrograde cholangiopancreatography (ERCP), which is widely utilised for both diagnosis and treatment.

Despite its effectiveness, ERCP is associated with post-procedural complications including acute pancreatitis, infections, gastrointestinal (GI) bleeds, and in rare cases perforations. An extremely rare but serious complication to be aware of is air embolism - occurring in around 3.32 out of 100,000 ERCP procedures [1].

Air embolism is a rare occurrence, often linked to vascular access surgeries, but it can also occur as a complication of ERCP. Mortality rates for ERCP-related air embolism are approximately 21%, with most of the cases resulting in fatal consequences within 48 hours after the event. Cardiac arrest is the most severe complication causing mortality in 53.8% of the individuals [2]. The volume and rate of gas accumulation, along with the patient's position during the event, are critical factors that influence outcomes. Here we present a case report of a 72-year-old female who experienced severe hemodynamic instability due to gas embolism during an ERCP procedure.

## Case presentation

A 72-year-old female, with a medical history of hypertension, iron-deficiency anaemia, vertigo and no past surgical history presented to the Accident and Emergency Department with a chief complaint of abdominal pain. An ultrasound confirmed the presence of multiple gall bladder stones, leading to a planned laparoscopic cholecystectomy, which proceeded without complications.

**Figure 1:**
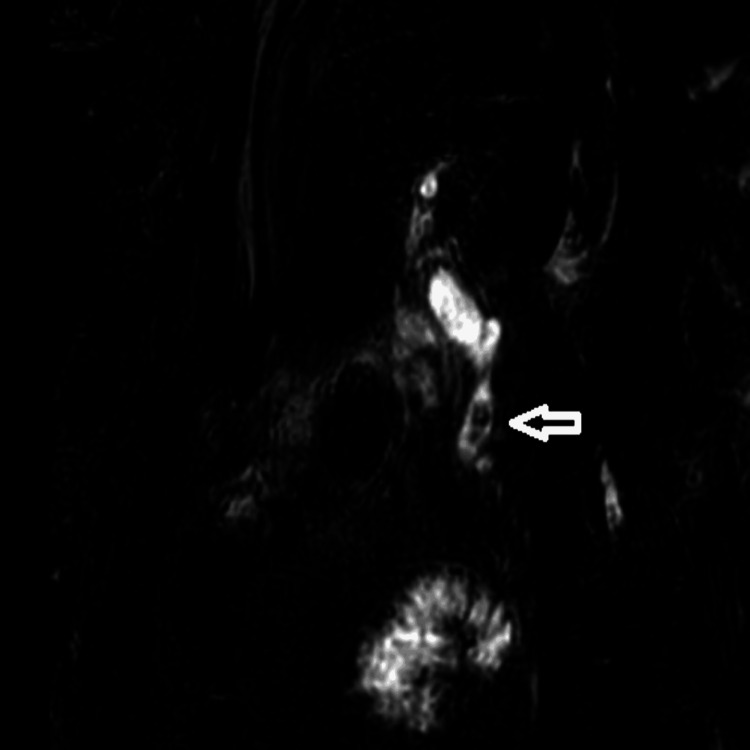
ERCP showing evidence of multiple common bile duct stones ERCP: Endoscopic retrograde cholangiopancreatography

Following the surgery, the patient continued to have a persistent pain in the abdomen and developed jaundice over the next few days. A follow-up magnetic resonance cholangiopancreatography (MRCP) showed an enlarged common bile duct with several stones, as observed in Figure 1. It was decided that this would be treated using endoscopic retrograde cholangiopancreatography.

**Figure 2:**
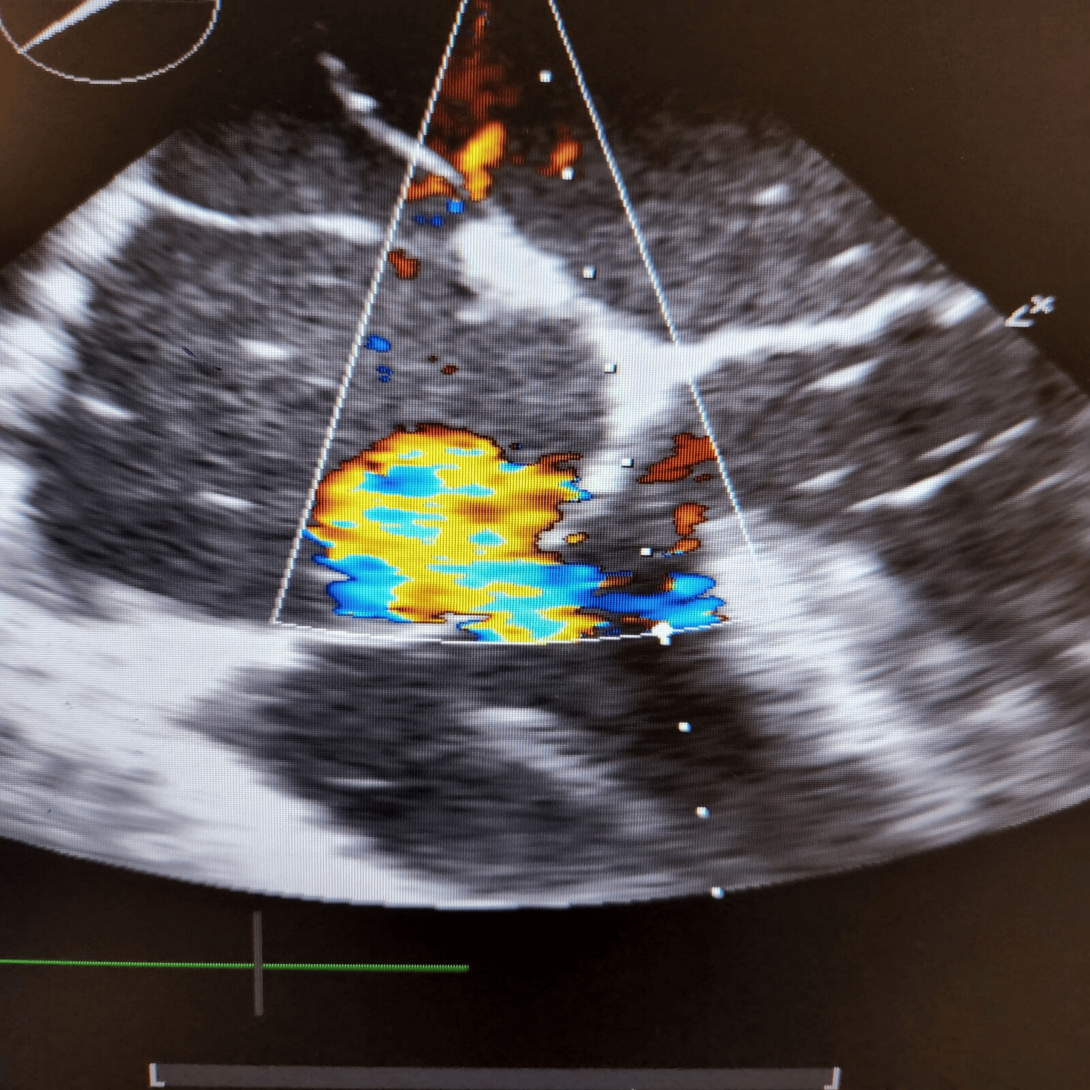
Gas bubbles seen in both the atria (with patent foramen ovale) in a 2D echo (echocardiogram)

**Figure 3:**
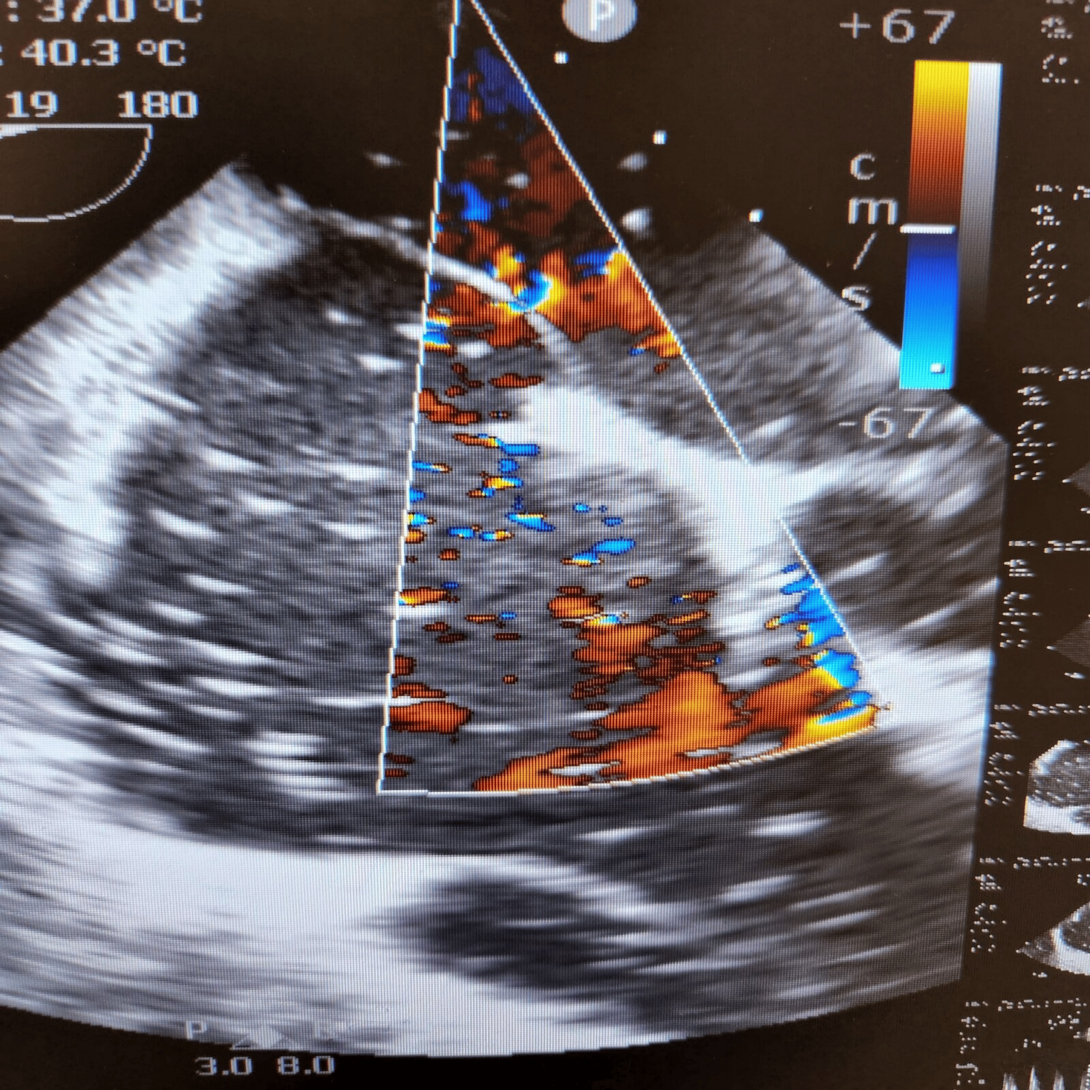
Gas bubbles seen in the left ventricle (seen as white spots) in a 2D echo (echocardiogram)

**Figure 4:**
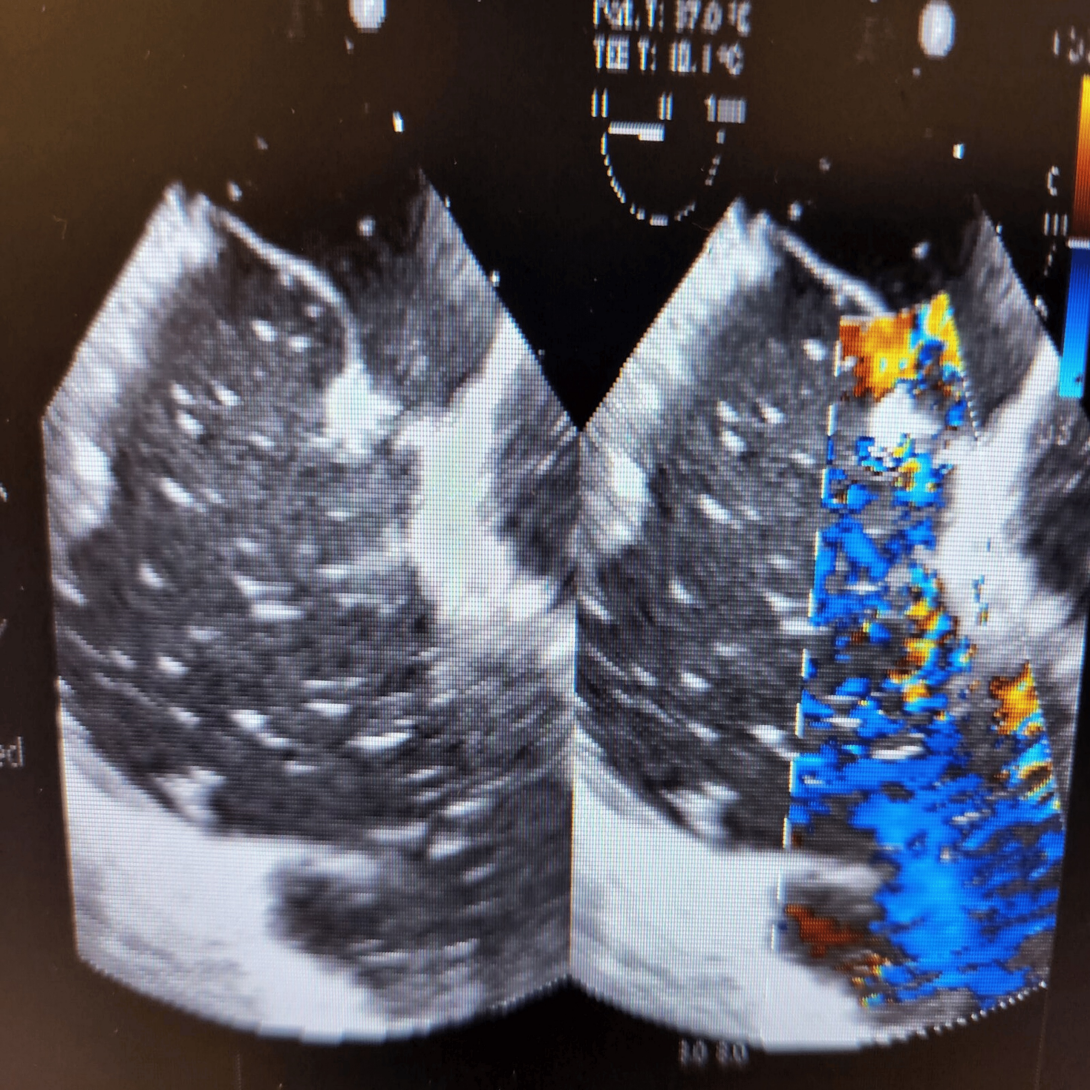
Gas bubbles seen passing through atrio-ventricular valves as seen in a 2D echo (echocardiogram)

During the final stages of the ERCP procedure, a significant decrease in oxygen saturation and the emergence of irregular heartbeats were noticed. An intraoperative trans-esophageal echocardiogram (echo) revealed numerous air pockets in the right atrium, right ventricle, and left ventricle. Importantly, a patent foramen ovale was detected as shown in Figure 2, with indications of air bubbles (seen as white spots on 2D echo) passing through it into the left side of the heart as seen in Figures 3, 4. The patient was intubated in response to decreased oxygen levels while still in the operating room.

Further management involved the administration of 100% oxygen, initiation of cardiopulmonary resuscitation, and immediate transfer to the intensive care unit for further treatment and monitoring [3,4].

Over the following days, her cardiovascular health worsened, requiring treatments like noradrenaline infusion and temporary pacemaker insertion. Regrettably, due to financial limitations, the patient chose to leave against medical advice despite being at great risk. The family received proper guidance regarding the patient's condition and related risks.

## Discussion

Gas embolism during ERCP is a rare but potentially fatal complication. It occurs when air enters the systemic circulation, leading to cardiovascular and pulmonary complications. One possible mechanism of gas embolism during ERCP is the intramural dissection of insufflated air into the portal venous system during endoscopic sphincterotomy [5]. In the presented case, the presence of a patent foramen ovale allowed for the passage of air bubbles from the right side of the heart to the left side, leading to systemic circulation and causing cardiovascular complications [2]. The patient's case is unique because it involves a cardiac air embolism leading to difficulties during ERCP, which is an uncommon complication. The presence of a patent foramen ovale further complicates the situation, as it allows for the passage of air bubbles into the left side of the heart, increasing the risk of systemic complications.

This case also highlights other difficulties, including the financial difficulties, which come with the private sector and insurance (which the patient had difficulty) as it requires explaining to the insurance companies how such pathologies can happen and getting the insured amount from them for the complications that are not highlighted in their documents!

## Conclusions

This case report contributes to the growing body of knowledge on ERCP-related air embolism, emphasising the need for heightened awareness and prompt management. The observed complications highlight the challenges in handling such rare occurrences, especially when financial considerations impact the course of medical care. By sharing this case, we aim to contribute valuable insights to the medical community and prompt further research into effective strategies for preventing and managing ERCP-related air embolism.
